# Use of Metadata-Driven Approaches for Data Harmonization in the Medical Domain: Scoping Review

**DOI:** 10.2196/52967

**Published:** 2024-02-14

**Authors:** Yuan Peng, Franziska Bathelt, Richard Gebler, Robert Gött, Andreas Heidenreich, Elisa Henke, Dennis Kadioglu, Stephan Lorenz, Abishaa Vengadeswaran, Martin Sedlmayr

**Affiliations:** 1 Institute for Medical Informatics and Biometry Carl Gustav Carus Faculty of Medicine Technische Universität Dresden Dresden Germany; 2 Thiem-Research GmbH Cottbus Germany; 3 Core Unit Datenintegrationszentrum University Medicine Greifswald Greifswald Germany; 4 Department for Information and Communication Technology (DICT), Data Integration Center (DIC) Goethe University Frankfurt, University Hospital Frankfurt am Main Germany; 5 Institute for Medical Informatics Goethe University Frankfurt, University Hospital Frankfurt Frankfurt am Main Germany

**Keywords:** ETL, ELT, Extract-Load-Transform, Extract-Transform-Load, interoperability, metadata-driven, medical domain, data harmonization

## Abstract

**Background:**

Multisite clinical studies are increasingly using real-world data to gain real-world evidence. However, due to the heterogeneity of source data, it is difficult to analyze such data in a unified way across clinics. Therefore, the implementation of Extract-Transform-Load (ETL) or Extract-Load-Transform (ELT) processes for harmonizing local health data is necessary, in order to guarantee the data quality for research. However, the development of such processes is time-consuming and unsustainable. A promising way to ease this is the generalization of ETL/ELT processes.

**Objective:**

In this work, we investigate existing possibilities for the development of generic ETL/ELT processes. Particularly, we focus on approaches with low development complexity by using descriptive metadata and structural metadata.

**Methods:**

We conducted a literature review following the PRISMA (Preferred Reporting Items for Systematic Reviews and Meta-Analyses) guidelines. We used 4 publication databases (ie, PubMed, IEEE Explore, Web of Science, and Biomed Center) to search for relevant publications from 2012 to 2022. The PRISMA flow was then visualized using an R-based tool (Evidence Synthesis Hackathon). All relevant contents of the publications were extracted into a spreadsheet for further analysis and visualization.

**Results:**

Regarding the PRISMA guidelines, we included 33 publications in this literature review. All included publications were categorized into 7 different focus groups (ie, medicine, data warehouse, big data, industry, geoinformatics, archaeology, and military). Based on the extracted data, ontology-based and rule-based approaches were the 2 most used approaches in different thematic categories. Different approaches and tools were chosen to achieve different purposes within the use cases.

**Conclusions:**

Our literature review shows that using metadata-driven (MDD) approaches to develop an ETL/ELT process can serve different purposes in different thematic categories. The results show that it is promising to implement an ETL/ELT process by applying MDD approach to automate the data transformation from Fast Healthcare Interoperability Resources to Observational Medical Outcomes Partnership Common Data Model. However, the determining of an appropriate MDD approach and tool to implement such an ETL/ELT process remains a challenge. This is due to the lack of comprehensive insight into the characterizations of the MDD approaches presented in this study. Therefore, our next step is to evaluate the MDD approaches presented in this study and to determine the most appropriate MDD approaches and the way to integrate them into the ETL/ELT process. This could verify the ability of using MDD approaches to generalize the ETL process for harmonizing medical data.

## Introduction

Multisite clinical studies are increasingly using real-world data to gain real-world evidence, especially during the COVID-19 pandemic [1]. However, not all clinics use the same hospital information system, resulting in heterogeneity of data produced by different hospital information systems. These heterogeneous data are not semantically and syntactically interoperable. Therefore, it is difficult to analyze such data in a unified way across sites. For this, the heterogeneous data need to be harmonized and standardized, for example, by using a common data model (CDM) [2]. For example, the European Medical Agency [3] set up the DARWIN EU (Data Analysis and Real World Interrogation Network European Union) [4] to provide real-world evidence on use and adverse events of medicines across the European Union. DARWIN EU uses the Observational Medical Outcomes Partnership (OMOP) CDM [5] as the base model, which is provided by the Observational Health Data Sciences and Informatics [6] community. To participate in such networks, a transformation of local data is needed. A common approach is to develop an Extract-Transform-Load (ETL) or Extract-Load-Transform (ELT) process. Both are used to harmonize heterogeneous data into the target systems. The only difference between them is the order of processing data. ETL transforms the data before loading them into the target systems, while ELT loads the data into the target systems first, and then transforms the data. Due to the different data formats and source systems, multiple ETL/ELT processes have to be implemented [7-10]. This work is time-consuming and hard to maintain [11].

Using a standard data exchange format can reduce the complexity of transforming heterogeneous data into CDMs. An example is the Fast Healthcare Interoperability Resources (FHIR) [12] format. FHIR is a communication standard and is provided by the Health Level 7 (HL7) [13]. In Germany, the Medical Informatics Initiative (MII) [14] provides a Core Data Set (CDS) [15] in FHIR format for enabling the interoperability of data across all university hospitals. Another German association “the National Association of Statutory Health Insurance Physicians” (KBV, German: Kassenärztliche Bundesvereinigung) [16] also provides a KBV CDS in FHIR format, which provides a stable foundation for the development of the medical information objects [17] (eg, immunization records and maternity records). Although both MII CDS and KBV CDS are based on the German HL7 Basis Profiles [18], the FHIR profiles defined in the 2 CDSs are not identical [19]. This is due to the different requirements of MII and KBV. For example, codes indicating departments within a clinic (eg, 0100 for internal medicine department) are defined in different value-sets and therefore use different coding systems. This also complicates the implementation and maintenance of ETL/ELT processes.

Furthermore, most countries try to standardize their electronic health records (EHR) data for research and to improve the interoperability of the data. Consequently, country-specific FHIR profiles are developed, for example, German HL7 Basis Profiles [18] and the US CDS [20]. Due to different languages (ie, German vs English), different structure definitions (eg, extensions and cardinality) and different coding systems (eg, system URL for International Classification of Diseases, 10, Revision: German Modification [21] vs system URL for International Classification of Diseases, 10, Clinical Modification [22]) used in the FHIR profiles, different ETL processes need to be implemented [8,23]. Although these are just a few examples, it is conceivable that with the expansion of supported use cases, the time required for implementing an ETL/ELT process increases massively, while the maintainability decreases. Therefore, the implementation of a generic ETL/ELT process for harmonizing local health data can guarantee the semantic and syntactic interoperability of research data across sites and countries.

Using metadata for the implementation of ETL/ELT processes is a promising approach, as stated by David Loshin [24]: “in order to organize data for analytical purposes, it will need to be extracted from the original source (source metadata), transformed into a representation that is consistent with the warehouse (target metadata) in a way that does not lose information due to differences in format and precision (structure metadata) and is aligned in a meaningful way (semantic metadata).” A very broad definition of metadata is “data about other data” [25]. Depending on the specific context of use, metadata can be classified into 3 types [26]:

- **Descriptive metadata**: the metadata is used for discovery and identification purposes, for example metadata for source and target data.
- **Structural metadata**: the metadata is used for managing data in information systems, for example, column names and table names in a database.
- **Administrative metadata**: the metadata exists within a database that provides additional information, for example, the name of a person, who has changed the data in a database.

Metadata can be represented by metadata languages (eg, Resource Description Framework and Notation3) [27]. Such languages are also called ontology languages. For enabling the interoperability of data from different source and target systems, rule languages (eg, Rule Markup Language and Semantic Web Rule Language) can be used to define the transformation rules between them [27]. Therefore, the use of metadata is expected to improve the development and maintenance for transforming FHIR resources to OMOP CDM.

As a side note, we understand any (descriptive and structural) metadata-based approach used for developing ETL/ELT processes as metadata-driven (MDD) approach. This work focuses on providing an overview of the types of MDD approaches and their use in different thematic categories. The overview aims to identify a suitable MDD approach to enhance the data transformation from FHIR to OMOP CDM. This will be achieved by answering the following questions:

- Q1: What are the themes of application for MDD approaches?
- Q2: What types of MDD approaches exist in the literature?
- Q3: What are the reasons for the usage of MDD approaches?
- Q4: What tool was used to implement the MDD approach?

## Methods

To answer our 4 research questions, we conducted a literature review. To ensure the transparency of the review process, we followed the PRISMA (Preferred Reporting Items for Systematic Reviews and Meta-Analyses) guidelines [28]. We used 4 publication databases (ie, PubMed, IEEE Explore, Web of Science, and Biomed Center) to search for relevant publications from 2012 to 2022 written in German or English (Textbox 1). The first search was performed on August 11, 2022, and the second one was on March 15, 2023, which in turn completed the search through December 31, 2022. The collected publications were loaded into the Zotero Citation Management program (Corporation for Digital Scholarship) [29] and the duplicates were manually removed. To better categorize the publications to be excluded, we defined 8 exclusion criteria (Textbox 2).

This review was a 2-fold process consisting of Title-Abstract-Screening (TAS) and full-text screening (FTS). Both screening processes used the same exclusion criteria listed in Textbox 2. The unique publications were divided into 2 groups based on their publication dates and uploaded to a research collaboration platform, Rayyan (Qatar Computing Research Institute and Cochrane Bahrain) [30], as 2 separate projects. Each publication group was assigned with 4 reviewers. The corresponding author reviewed all publications. The TAS was performed under the blind-modus, so that each reviewer could label the publication independently. The blind-modus was turned off after all publications were tagged and the conflicts were discussed and resolved. After that, all included publications were randomly divided into 2 groups and reloaded into Rayyan as a new project for FTS. Similar to TAS, 4 reviewers were assigned to each publication group and the corresponding author reviewed all publications. The FTS was also conducted under the blind-modus and followed the same review process as the TAS.

We extracted the content of all included publications based on the categories listed in Textbox 3. The extraction of publication content was done by the corresponding author and validated by 4 coauthors. The extracted content was stored in a spreadsheet for further analysis and visualization.

The result of the literature review was visualized using an R-based tool, which was developed based on PRISMA 2020 [31].

**Search string and publication databases.**

Search string

**PubMed**

- ((meta data) OR (meta-data) OR (metadata) OR (ontology) OR (rules)) AND ((extract transform load) OR (ETL) OR (extract load transform) OR (ELT))

**IEEE Explore**

- ((“All Metadata”:metadata) OR (“All Metadata”:meta-data) OR (“All Metadata”:meta data) OR (“All Metadata”:ontology) OR (“All Metadata”:rules)) AND ((“All Metadata”:ETL) OR (“All Metadata”:extract transform load) OR (“All Metadata”:ELT) OR (“All Metadata”:extract load transform))

**Web of Science**

- (ALL=(metadata) OR ALL=(meta-data) OR ALL=(“meta data”) OR ALL=(ontology) OR ALL=(rules)) AND (ALL=(ETL) OR ALL=(“extract transform load”) OR ALL=(ELT) OR ALL=(“extract load transform”))

**Biomed Center (BMC)**

- (“meta data” OR meta-data OR metadata OR ontology OR rules) AND (“extract transform load” OR ETL OR “extract load transform” OR ELT)

**Labels and descriptions of exclusion criteria.**

**Wrong_abbreviation**

- Publication does not contain Extract-Transform-Load (ETL) as “Extract-Transform-Load.”
- Publication does not contain Extract-Load-Transform (ELT) as “Extract-Load-Transform.”

**Wrong_definition**

- Publication does not use metadata in the context of “metadata of data in source or target.”
- Publication does not use rules in the context of “rules for data transformation.”

**Only_etl_elt**

- Publication describes only ETL/ELT.

**Only_metadata**

- Publication describes only metadata.

**Wrong_focus**

- Publication mentioned metadata and ETL/ELT, but the focus is not about data harmonization

**Wrong_type**

- Publication is not a conference paper or a journal publication

**Foreign_language**

- Publication is written in other languages than English and German

**Wrong_content**

- Publication does not mention ETL/ELT or metadata

**Categories for data extraction.**

**Theme**

- The main theme of the work.

**Metadata-driven method**

- The used metadata-driven method in the work.

**Metadata-driven method tool**

- Tool which was used to conduct the metadata-driven method.

**Purpose**

- The purpose of using the metadata-driven method.

## Results

### Literature Search

**Figure 1:**
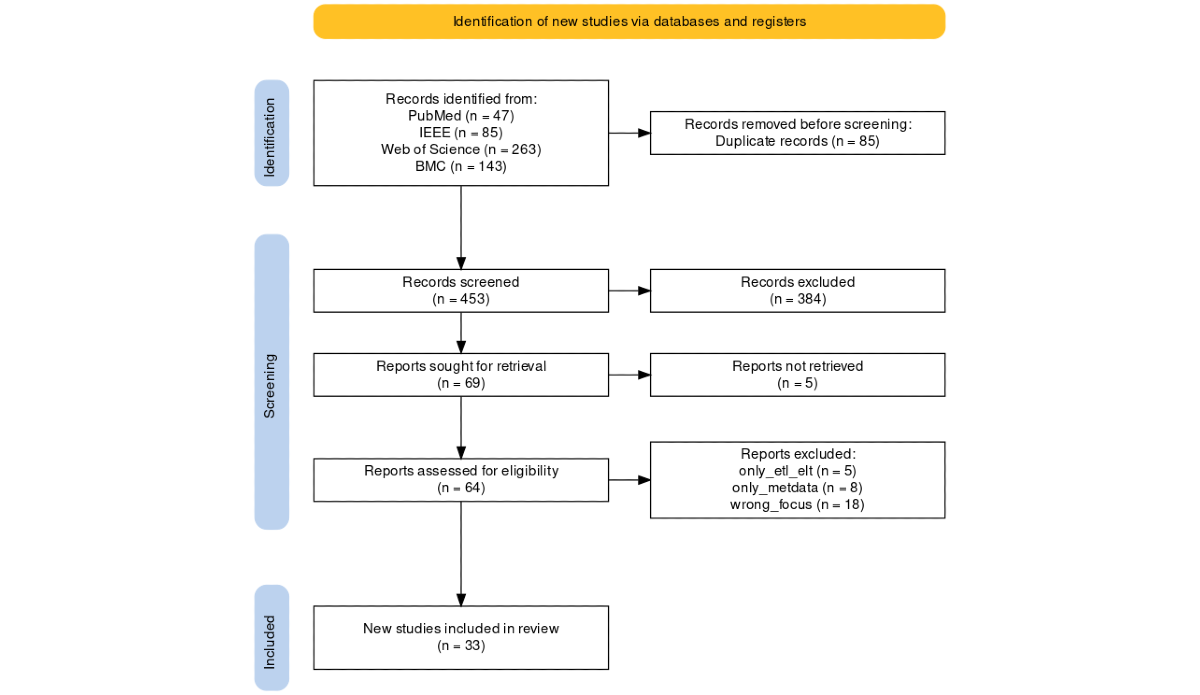
PRISMA (Preferred Reporting Items for Systematic Reviews and Meta-Analyses) flow diagram. Generated using an R-based tool (reproduced from Haddaway et al [31], with permission from Neal R Haddaway).

The literature search resulted in 538 publications. After removing 85 duplicates, 453 publications were screened during the TAS phase. By using the exclusion criteria defined in Textbox 2 and excluding the publications, which have no full-text, 64 publications were included for FTS. Finally, we included 33 publications in this work. The screening process and results are structured using the PRISMA flow diagram 2020 (Figure 1). A complete list of included publications is available in Multimedia Appendix 1.

### Distribution of Publications

In order to gain an overview of the potential application focuses of MDDs (Q1) and thus an indication of where the approaches have proven beneficial, the focused theme of application was first evaluated. According to the extracted data, the focuses of all included publications are classified into 7 different categories, namely medicine (n=9) [10,32-39], data warehouse (n=13) [40-52], big data (n=4) [53-56], industry (n=4) [57-60], geoinformatics (n=1) [61], archaeology (n=1) [62], and military (n=1) [63]. This shows that data warehouse and medicine are the 2 categories that use the MDD approach the most.

### MDD Approaches Used for Various Thematic Categories

**Figure 2:**
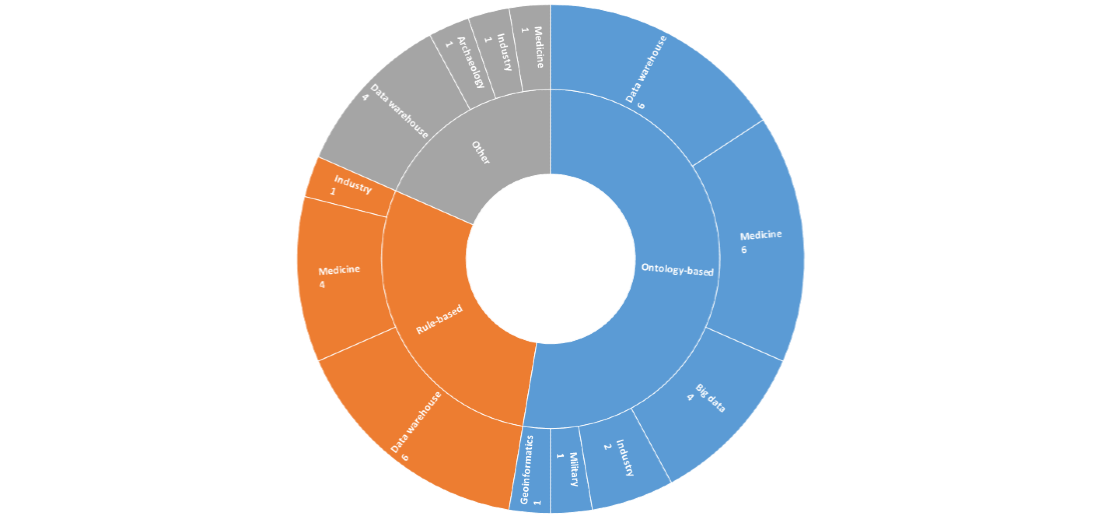
Metadata-driven approaches used in each thematic category.

Different types of MDD approaches were used across the thematic categories. To gain knowledge about the use of these types of MDD approaches in each category (Q2), the distribution of MDD approaches was investigated. Figure 2 shows the application of different types of MDD approaches in different thematic categories. The most frequently used type of MDD approach was ontology-based, where the ontology (using for example, resource description framework) of the source or target was applied in the ETL/ELT process. This approach was used in 6 categories, particularly in the categories of data warehouse [45-48,50,52] and medicine [10,32,35,37-39]. Another frequently used type of MDD approach was rule-based, which applied transformation rules generated based on the source and target to the ETL/ELT process. The rule-based approach was also widely used in the categories of data warehouse [40-43,49] and medicine [33,34,37,39]. All other MDD approaches besides the ontology-based and rule-based approaches were categorized as “other” (Table 1).

**Table 1:** MDD^a^ approaches that are categorized as “other.”

MDD approach type and publication		Example
**UML^b^-based**		
	Dhaouadi et al [46]	UML class diagram is used for modeling the transformation process
**Graphic-based**		
	Dhaouadi et al [46]	BPMN^c^ standard is used for modeling an ETL^d^ process
**Ad hoc formalisms-based**		
	Dhaouadi et al [46]	Entity Mapping Diagram is used for representing ETL tasks
**MDA^e^-based**		
	Dhaouadi et al [46]	MDA is a multilayered framework with multiple submodules for separation of the specification of a functionality from its implementation
**Message-based**		
	Novak et al [51]	“Normal message” contains information of mapping and transformation; “command message” configures the (execution) system
**Template-based**		
	McCarthy et al [58]	A transformation template for each data source that manages the complex transformation process
	Binding et al [62]	A template contains the mapping patterns which is then used for querying in database
**Metadata-based^f^**		
	Ozyurt and Grethe [36]	Implementing a generic data transformation language to transform heterogeneous data from multiple sources to a common format
	Tomingas et al [44]	Metadata of the source and target stored in a knowledge and metadata repository
	Suleykin and Panfilov [60]	Metadata of the mapping path stored in a metadata management framework

^a^MDD: metadata-driven.

^b^UML: unified modeling language.

^c^BPMN: Business Process Model Notation.

^d^ETL: Extract-Transform-Load.

^e^MDA: Model Driven Architecture.

^f^Metadata-based approach: approach uses metadata without any specification.

### Purposes of Using MDD Method for Data Harmonization

**Figure 3:**
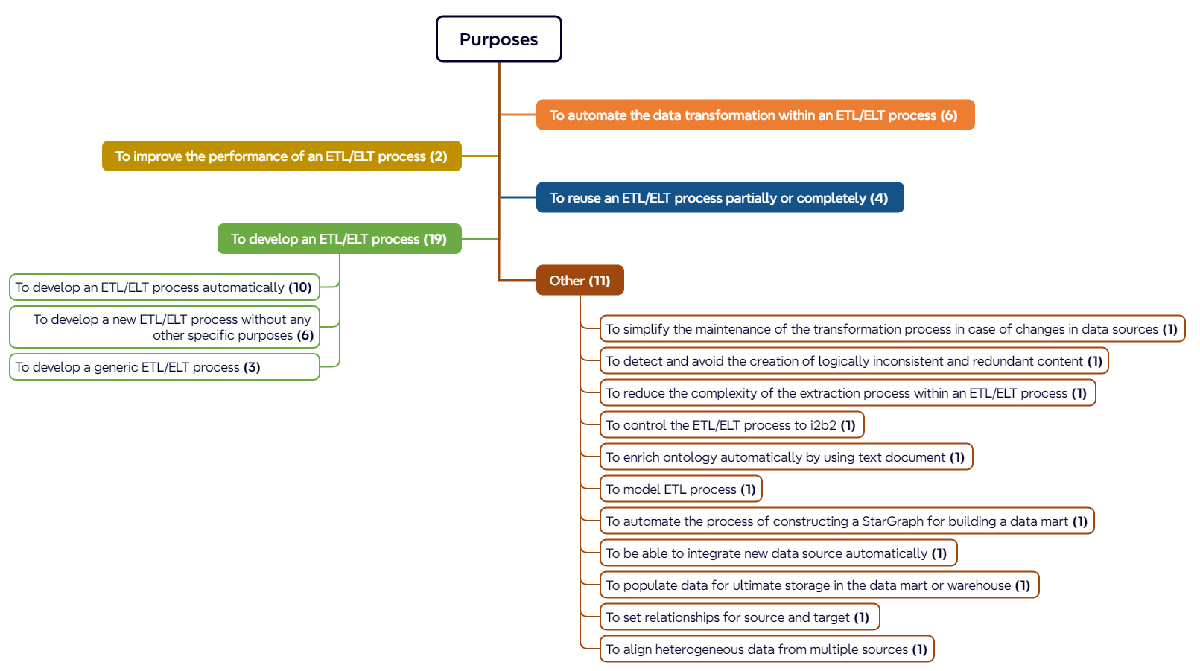
Purposes of using MDD approaches in ETL/ELT process. ELT: Extract-Load-Transform; ETL: Extract-Transform-Load; i2b2: Informatics for Integrating Biology and the Bedside; MDD: metadata-driven.

The purpose of using MDD approaches in each use case was then investigated to clarify the reasons why MDD approaches were used (Q3). Figure 3 shows different purposes of using MDD approaches in developing ETL/ELT processes based on the extracted data. The majority of publications describe the use of MDD approaches to develop an ETL/ELT process. This purpose can be divided into three detailed categories: (1) to automate the development of the ETL/ELT process [35,38,42,46,48-51,60], (2) to develop a generic ETL/ELT process [39,47,52], and (3) to develop a new ETL/ELT process without any further technical specifications [40,45,46,55,57,61]. Additionally, the transformation part of the ETL/ELT process could also be automated by applying an MDD approach [34,37,41,44,58,63]. For example, Chen and Zhao [41] described an MDD approach for the automatic generation of SQL scripts for data transformation. Moreover, using MDD approaches can also help to improve the performance of ETL/ELT processes [43,46] or to partially or fully reuse the ETL/ELT process [10,33,43,62]. Other goals (categorized as “Others” in Figure 3), such as simplifying the maintenance of the transformation process [37] and reducing the complexity of the extraction process [53], can also be realized by using MDD approaches in ETL/ELT processes.

### Relationship Between Use Case and Used MDD Approach

As shown in the previous section, different MDD approaches were applied for different purposes. To further elucidate the reasons for choosing MDD approaches (Q3), the relationship between them was investigated. Table 2 lists the number of publications, which used a type of MDD approach to achieve a specific purpose. The ontology-based approach was used to achieve purposes (1) and (2), and (4)-(7). For example, Huang et al [63] created both local ontology (ontology based on the source data) and global ontology (ontology for the query processing) for the data transformation process, so that the data transformation from local ontology to global can be automated by applying ontology learning, ontology mapping, and ontology rules. Additionally, the ontology-based approach was also used to achieve other goals, such as controlling the ETL process to Informatics for Integrating Biology and the Bedside [32] and reducing the complexity of the extraction process [53]. Similar to the ontology-based approach, the rule-based approach was used to achieve the purposes of (1)-(3) and (5)-(7). Due to the reusability of the transformation rules, it was also possible to simplify the maintenance of the ETL/ELT process by applying rules in the process [37]. Other MDD approaches such as template-based [58,62], message-based [51], and metadata-based [41,44,48] were used to achieve the goals of (1)-(3) and (5)-(7). A metadata-based approach (eg, metadata management framework) can be used to develop the ETL tasks automatically [60]. The detailed information of Table 2 is available in the Multimedia Appendix 1.

**Table 2:** Relationships between purposes and MDD^a^ approaches used.

Purposes		MDD approaches		
Number	Description	Ontology-based, n/N (%)	Rule-based, n/N (%)	Other, n/N (%)
(1)	To automate the data transformation within an ETL^b^/ELT^c^ process	2/6 (33)	3/6 (50)	1/6 (17)
(2)	To reuse an ETL/ELT process (partially or completely)	1/4 (25)	2/4 (50)	1/4 (25)
(3)	To improve the performance of an ETL/ELT process	0/2 (0)	1/2 (50)	1/2 (50)
(4)	To develop a generic ETL/ELT process	3/3 (100)	0/3 (0)	0/3 (0)
(5)	To develop an ETL/ELT process automatically	5/9 (56)	2/9 (22)	2/9 (22)
(6)	To develop a new ETL/ELT process (without any other specific purposes)	4/6 (67)	1/6 (17)	1/6 (17)
(7)	Other	5/11 (45)	2/11 (18)	4/11 (36)

^a^MDD: metadata-driven**.**

^b^ETL: Extract-Transform-Load**.**

^c^ELT: Extract-Load-Transform**.**

### Tools Used for Implementing MDD Approaches

**Figure 4:**
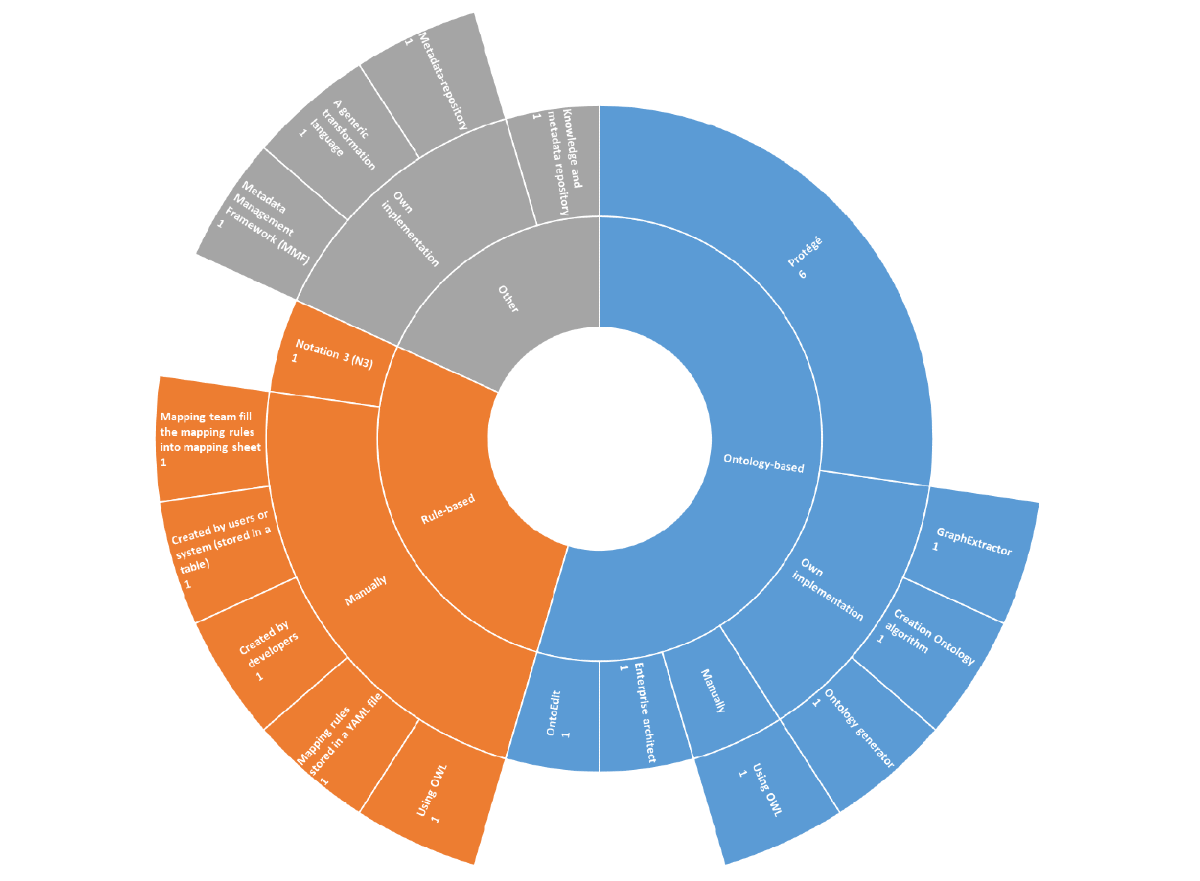
Tools used for developing the metadata-driven approach. MMF: metadata management framework; OWL: Web Ontology Language; YAML: YAML Ain’t Markup Language.

Finally, we focused on the tools used to implemented MDD approaches (Q4). For achieving various purposes as shown in the previous section, different tools were used. As shown in Figure 4, each type of MDD approach can be implemented by using either an existing tool or a use case specific tool. Based on the included publications, the ontology-base approaches were mostly implemented using Protégé (Stanford Center for Biomedical Informatics Research) [64]. Protégé is an ontology editor, as well as OntoEdit (Institute AIFB, University of Karlsruhe and Ontoprise GmbH) [65]. The main reason for using an ontology editor is its ease of use and maintenance, as well as the various plug-ins. The use of case specific tools, such as ontology generator introduced by Kamil et al [45], generated ontologies based on the data definition language of the relational database. Both types of tools were used for creating and maintaining the ontology, which was then used to establish a generic mapping logic in the ETL/ELT process [32,50,52,54,55,61]. Another type of frequently used MDD approach is rule-based, which is used for phrasing and storing the transformation rules. The transformation rules can be stored in a mapping sheet [49], a CSV file [34], a YAML (YAML Ain’t Markup Language) file [33] or a table within a database [43], which were implemented manually. Afterwards, the transformation rules could be used in the ETL/ELT process, for example, to enable the automatic transformation. Other types of MDD approaches can also be implemented by using existing tools (eg, knowledge and metadata repository [66]) or use case specific tools (eg, metadata repository [41] and metadata management framework [60]). For example, Ozyurt and Grethe [36] implemented a generic transformation language using the bioCADDIE Data Tag Suite (bioCADDIE Project) [67] (a metadata schema) to align heterogeneous data from multiple sources, which provided a basis for further analytic queries.

## Discussion

### Principal Findings

Our literature review on the topic “metadata-driven ETL/ELT” includes all publications listed on PubMed, IEEE Explore, Web of Science, and Biomed Center on MDD ETL/ELT process from 2012 to 2022. In some context, the use of metadata is represented specifically using “ontology” or “rules.” Therefore, we added “ontology” and “rules” into the search string to expand the search range.

With the review process presented, we were able to provide an overview of the thematic categories to which the MDD ETL/ELT processes were applied (Q1), the types of MDD approaches used in the ETL/ELT processes (Q2), the purposes of using MDD approaches (Q3), as well as the tools used to implement the MDD approaches (Q4).

Across all thematic categories, ontology-based and rule-based approaches are the most used approaches in the data warehouse and the medical thematic categories. In some cases, more than one MDD approach was used in the ETL/ELT process. For example, Del Carmen Legaz-García et al [39] used both ontology-based and rule-based approaches. Therefore, such publications were categorized as both MDD approach types.

Various tools can be used to implement MDD approaches. Unfortunately, we were not able to extract this information from all included publications. The reason for that is that some publications used proprietary or nontransferable approaches (eg, data-specific ontologies [39,62] and rules from Data Vault [DataVaultAlliance] [42]). Some other publications did not explicitly mention or describe the tools they used. Therefore, these publications were not included in the analysis of MDD tools used.

The results indicate that it is promising to implement a generic ETL/ELT process to transform different FHIR profiles to OMOP CDM automatically by applying MDD approaches. However, the results do not provide a trivial solution for this. For example, Huang et al [63] used an ontology-based approach to be able to automate the data transformation in an ETL/ELT process, while Ong et al [34] used a rule-based approach to achieve the same purpose. In some cases, more than one MDD approach were used as complements in order to accomplish the data transformation. For example, Pacaci et al [37] chose an ontology-based approach to automate the data transformation and a rule-based to simplify the maintenance of the transformation process in case of changes in data sources. By applying these 2 approaches in combination, the authors were able to transform EHR data from heterogeneous EHR systems into OMOP CDM. Therefore, determining an appropriate MDD approach and tool to implement a generic ETL/ELT process to transform FHIR to OMOP CDM automatically remains a challenge.

This work aimed to provide an overview of different types of MDD approaches and their tools. Consequently, this review lacks an analysis of detailing the specific traits of each MDD approach. This gap underscores the importance of providing a comprehensive insight into the characterizations of the MDD approaches presented in this study. This analysis will be conducted in the future to provide solid evidence for selecting the most suitable MDD approach and tool, or for considering using multiple MDD approaches in combination to implement the generic ETL/ELT process for transforming FHIR to OMOP CDM.

### Conclusions

Our literature review shows that using MDD approaches to develop an ETL/ELT process can serve different purposes in different focus groups (ie, medicine, data warehouse, big data, industry, geoinformatics, archaeology, and military). The results show that it is promising to implement an ETL/ELT process by applying MDD approach for automating the data transformation from FHIR to OMOP CDM. However, the determination of an appropriate MDD approach and tool to implement such an ETL/ELT process remains a challenge. This is due to the lack of comprehensive insight into the characterizations of the MDD approaches presented in this study. Therefore, our next step is to evaluate the MDD approaches presented in this study and to determine the most appropriate MDD approaches and the way of integrating them into the MII CDS FHIR to OMOP CDM ETL process [8]. This could verify the ability of using MDD approaches to generalize the ETL process for harmonizing medical data [11].

## Appendix

Multimedia Appendix 1 Excel tables for extracted data from included publications.

Multimedia Appendix 2 PRISMA-ScR checklist.

## Abbreviations

CDM: Common Data Model
CDS: Core Data Set
DARWIN EU: Data Analysis and Real World Interrogation Network European Union
EHR: electronic health record
ELT: Extract-Load-Transform
ETL: Extract-Transform-Load
FHIR: Fast Healthcare Interoperability Resources
FTS: full-text screening
HL7: Health Level 7
KBV: The National Association of Statutory Health Insurance Physicians (German: Kassenärztliche Bundesvereinigung)
MDD: metadata-driven
MII: Medical Informatics Initiative
OMOP: Observational Medical Outcomes Partnership
PRISMA: Preferred Reporting Items for Systematic Reviews and Meta-Analyses
TAS: Title-Abstract-Screening
YAML: YAML Ain’t Markup Language
